# Analysis of microbial structure and function in fermented grains during the fermentation process of Congjiang WeiJiu based on high-throughput sequencing

**DOI:** 10.7717/peerj.21180

**Published:** 2026-05-21

**Authors:** Lei Shao, Shucai Li, Long Yang, Shenbing Wei, Gang Shen, Lihong Shi, Jiahong Zhu, Bohan Ding, Yu Liu, Yuxin Shi, Yong Liu

**Affiliations:** 1 College of Pharmacy, Guiyang Healthcare Vocational University, Guiyang, Guizhou, China; 2 Guizhou Provincial Engineering Research Center of Medical Resourceful Health Care Products, Guiyang Healthcare Vocational University, Guiyang, Guizhou, China; 3 Guizhou Taibang Biological Products Co., Ltd., Guiyang, Guizhou, China; 4 Qiandongnan Miao and Dong Autonomous Prefecture, Guizhou Congjiang Western Zhuang Weijiao Liquor-Making Co., Ltd., Qiandongnan Miao and Dong Autonomous Prefecture, Guizhou, China; 5 Guiyang Healthcare Vocational University, College of Smart Elderly Care and Modern Housekeeping, Guiyang, Guizhou, China

**Keywords:** WeiJiu, Fermentation processes, Microbial community structure, Diversity, Functional annotations, High-throughput sequencing

## Abstract

**Background:**

WeiJiu was a traditional specialty liquor from the Zhuang ethnic villages in Congjiang County, Qiandongnan Miao and Dong Autonomous Prefecture, Guizhou Province. It was brewed using glutinous Xianghe rice, mountain spring water, and ancestral *koji* as raw materials. Its core production processes consist of five stages: (1) raw material preparation; (2) spreading, cooling and yeast mixing; (3) fermentation and liquor extraction; (4) simmering treatment; (5) sealing and aging. WeiJiu had a dark brown color, a mellow, soft, and sweet taste, and featured the characteristic of becoming more aromatic as it ages. As an intangible cultural heritage item of Qiandongnan Prefecture, its craftsmanship inheritance had long been confined to an empirical paradigm. Due to the lack of research on the composition and function of the microbial community in Congjiang WeiJiu, the microbial changes and metabolite changes during the fermentation process, its quality characteristics and brewing mechanism remain unclear. Therefore, in-depth understanding of the brewing mechanism and essentially improving its quality and production was an urgent priority for research related to Congjiang WeiJiu.

**Method:**

In this study, the fermented grains of Congjiang WeiJiu at various fermentation stages: CQ: early fermentation stage (7 d), ZQ: middle fermentation stage (11 d) and WQ: late fermentation stage (15 d) were used as the research objects. High-throughput sequencing technology was employed to analyze microbial community structure and diversity. Functional annotations were performed against KEGG and CAZys databases to explore metabolic pathways and carbohydrate-active enzyme (CAZys) characteristics.

**Results:**

The microbial community exhibited significant stage-specific succession synchronized with fermentation processes. At the phylum level, Bacillota and Pseudomonadota dominated in CQ, Bacillota became predominant in ZQ, and Actinomycetota increased significantly in WQ. At the genus level, *Aspergillus*, *Saccharomyces*, and *Hyphopichia* served as core functional genera in respective stages. Functional annotations showed stage-specific expression of metabolic pathways: KEGG pathways focused on energy and amino acid metabolism (in CQ), carbohydrate metabolism (in ZQ), and stress adaptation (in WQ). CAZys families corresponded to fermentation substrates degradation (GH28, AA1 in CQ), macromolecule conversion (GH13, CBM50 in ZQ), and metabolite modification (GH18, GH16 in WQ). Core functional bacteria enhanced adaptability through evolutionary mechanisms such as horizontal gene transfer, genome streamlining, and plasmid-mediated gene acquisition. The unique simmering process and smoked cellar storage shaped the distinct microbial community and flavor, differing from traditional Luzhou-flavor liquor in yeast succession, lactic acid bacteria metabolism, and mold survival period.

## Introduction

Congjiang WeiJiu, a provincial intangible cultural heritage of Guizhou, originated from Congjiang County, Qiandongnan Miao and Dong Autonomous Prefecture. Brewed by integrating traditional and modern techniques, it used local fragrant glutinous rice and mountain spring water as raw materials, undergoing raw material processing, spreading for cooling and *koji* mixing, fermentation and liquid extraction, unique gentle simmering (a core process endowing it with a rich, smooth texture and long-lasting aroma), and sealed aging, while adhering to ecological brewing principles. However, Congjiang WeiJiu has long relied on family-style inheritance and workshop-scale production, with quality depending on brewers’ empirical experience rather than scientific standards, resulting in unstable product quality. Currently, only research on its production process has been reported (Xie et al., 2024), while studies on its *koji*, fermentation process, and microbial community composition remain scarce. The lack of understanding of microbial and metabolite dynamics during fermentation had left its brewing mechanism and quality characteristics unclear, making in-depth exploration of the brewing mechanism crucial for quality improvement and standardized production.

High-throughput sequencing technology, characterized by a wide detection range and high sensitivity to low-abundance microorganisms, has been widely applied in food and fermented alcoholic beverage research-especially for starch-based matrices (*e.g*., rice, corn, wheat, beans; Wang et al., 2025, Wu et al., 2019). This technology had revolutionized the analysis of microbial community diversity, dynamic changes, and their correlation with flavor formation in fermented systems. For starch-rich seed fermentations, relevant studies have provided valuable insights. Hu et al. (2020) analyzed microbial succession in strong-flavor Baijiu fermented grains (solid-state fermentation with sorghum as the main starch raw material. Ma et al. (2020) identified 310 bacterial genera and 59 fungal genera in Laobaigan-type Baijiu brewing (a typical northern Chinese Baijiu produced *via* solid-state fermentation using sorghum as the core starch raw material. Ji et al. (2018) studied fungal dynamics in Huangjiu fermentation (semi-solid fermentation with glutinous rice as the main starch source) using Illumina-Miseq technology, finding *Aspergillus* as the dominant genus across all stages, suggesting its metabolites contribute to key chemical components of Huangjiu. Chen et al. (2023) analyzed the fermented mash of Shaoxing mechanized Huangjiu (a representative semi-solid fermentation product of East China, with glutinous rice as the primary starch raw material) *via* Illumina-HiSeq, identifying dominant microorganisms including *Saccharomyces*, *Lactococcus*, and *Lactobacillus*. Studying the diversity of microorganisms in fermented grains was helpful for optimizing fermentation processes and better in-depth analysis of the relationship between microbial communities and quality of fermented alcoholic beverage.

Fermented grains from different fermentation stages of Congjiang WeiJiu as the research object, and high-throughput sequencing technology was used to analyze the community structure and microbial functions of WeiJiu in this study. The aim of this study was to clarify the microbial community composition, dynamic changes and succession trend during the brewing of Congjiang WeiJiu, thereby providing certain theoretical support for revealing the fermentation mechanism and improving the quality of Congjiang WeiJiu.

## Materials AND Methods

### Experimental materials

#### Materials

Fermentation samples utilized in this study consisted of fermented grains collected at different fermentation stages from the fermentation tanks of Congjiang Xizhuang WeiJiu Brewing Co., Ltd., located in Congjiang County, Qiandongnan Miao and Dong Autonomous Prefecture, Guizhou Province, China. The brewing process adopted solid-liquid mixed fermentation method. The main reagents used were as follows: Taq deoxyribonucleic acid (DNA) polymerase (5 U/μL) from Thermo Fisher Scientific (Waltham, MA, USA); E.Z.N.A. Soil DNA Extraction Kit from OMEGA (Norcross, GA, USA); SanPrep Column DNA Gel Extraction Kit from Sangon Biotech (Shanghai) Co., Ltd.; Qubit 2.0 DNA Assay Kit from Life (Carlsbad, CA, USA). All other reagents such as Proteinase K, Anhydrous Ethanol Agarose, were of analytical grade and domestically produced.

### Experimental methods

#### Sample collection

The type of fermentation of Weijiu was characterized as a composite traditional technique, involving initial solid-state culture and saccharification, followed by liquid/semi-solid anaerobic fermentation, and finalized with a unique post-fermentation heat treatment (stewing) for termination and maturation. According to the brewing process of Congjiang WeiJiu, samples were collected on days 7, 11, and 15 of fermentation, which were designated as the early (CQ), middle (ZQ), and late (WQ) fermentation stages, respectively. For each time point, equal amounts of samples were taken from three different heights (top, middle, and bottom) of the fermentation tank and mixed thoroughly to ensure sample representativeness. After collection, samples were immediately frozen in liquid nitrogen and then stored at −20 °C until DNA extraction. All extractions were completed within 2 weeks, and all experiments were performed with three independent biological replicates.

#### Extraction of DNA

Total genomic DNA of microorganisms from the fermented grains at different fermentation stages sample was extracted using the E.Z.N.A. Soil DNA Kit. The extraction steps included sample pretreatment, cell lysis, centrifugation to collect the supernatant, nucleic acid binding, washing, column membrane drying, and DNA elution. The purity (OD 260/280 ratio) and integrity of the DNA were detected by 1% agarose gel electrophoresis and Nanodrop, while the concentration of the DNA was accurately quantified using Qubit.

#### Construction and quality control of the library

Genomic DNA (1 μg) was randomly fragmented to approximately 350 bp using a Covaris ultrasonic disruptor, and sequencing libraries were constructed *via* end repair, A-tailing, adapter ligation, purification, and PCR amplification. Library integrity and insert size were examined using an AATI (Agilent Bioanalyzer 5400 system) device. Libraries with acceptable quality were quantified by quantitative real-time polymerase chain reaction (q-PCR), effective concentration >3 nM) and pooled according to effective concentration and target data volume, followed by PE150 sequencing.

#### Data processing

Scaftigs were generated after assembling the effective data (Clean Data) obtained by filtering the raw data. Based on the assembled scaftigs, gene prediction and redundancy removal were performed to construct a gene catalogue, which was then used to calculate the abundance information of the corresponding gene catalogue across different samples. Metagenomic assembly, species annotation, and abundance analysis were performed on the data.

#### Analysis of α diversity

Alpha diversity comparison and analysis were conducted on the microorganisms in fermented grains at different fermentation stages, including the Shannon index, Simpson index, Ace index, Chao1 index, and coverage. Species richness was reflected by Chao1, Observed_species, and ACE values, while the Shannon index and Simpson index reflected the evenness of species diversity. One-way ANOVA combined with Tukey’s HSD *post-hoc* test was used to analyze the differences among groups, with statistical significance defined as *p* < 0.05 (significant difference) and **p < 0.001 (highly significant difference).

### Bioinformatic analysis

Starting from the gene catalogue, functional annotation was conducted against the KEGG database (Kyoto Encyclopedia of Genes and Genomes, KEGG) and the CAZys Database (Carbohydrate-Active Enzymes Database, CAZys).

## Results

### Data analysis

Genomic DNA extracted was subjected to high-throughput sequencing. The valid sequencing data (Clean Data) were assembled into scaftigs, and statistical analysis was performed on the assembled scaffold characteristics, with the results presented in Table 1. In CQ, a total of 424,026 valid sequences were obtained, with an average length of 891. In ZQ, a total of 181,422 valid sequences were obtained, with an average length of 1,176. In WQ, a total of 261,122 valid sequences were obtained, with an average length of 995 (Table 1). In WQ, the number of scaftigs decreased by 57% compared with the early stage, indicating that the composition of the microbial community in the fermented grains was the most abundant in the early stage of fermentation, and the structure of the microbial community tended to be simplified with the extension of fermentation time. The N50 of the ZQ sample was much higher than that of the CQ sample in Congjiang WeiJiu, indicating that the genome continuity of the microbial community in the mid-fermentation stage was better, and it was inferred that the dominant bacteria were prominent in the mid-fermentation stage. The N90 of WeiJiu at each stage was around 540 bp, suggesting that there were microbial communities with high complexity in the fermented grains samples at each stage.

**Table 1:** Statistics of metagenomic high-throughput sequencing assembly data for microbial communities in Congjiang WeiJiu fermented grains at different fermentation stages.

Sample ID	Total length (bp)	Scaftigs numbers	Average length (bp)	N50 length (bp)	N90 length (bp)	Max length (bp)
CQ1	386,755,827	432,427	894.38	786	537	347,485
CQ2	376,884,653	423,333	890.28	786	538	295,301
CQ3	370,361,574	416,319	889.61	784	537	369,554
ZQ1	207,417,535	174,787	1,186.69	1,209	555	316,556
ZQ2	229,643,474	199,997	1,148.23	1,129	554	316,526
ZQ3	202,363,143	169,482	1,194.01	1,240	556	334,220
WQ1	239,868,869	234,940	1,020.98	862	541	348,720
WQ2	271,168,059	276,100	982.14	831	540	457,416
WQ3	267,676,093	272,327	982.92	829	540	316,521
CQ_m	378,000,685	424,026	891.42	785	537	337,447
ZQ_m	213,141,384	181,422	1,176.31	1,193	555	322,434
WQ_m	259,571,007	261,122	995.35	841	540	374,219

**Notes:**

CQ, early fermentation stage (7 d); ZQ, middle fermentation stage (11 d); WQ, late fermentation stage (15 d).

The numbers 1–3 indicated three biological replicates.

m, Average value of three biological replicates.

### Analysis of α diversity of microbial communities in fermented grains at different fermentation stages

One-way analysis of variance (ANOVA) combined with Tukey’s HSD *post-hoc* test was used to analyze the differences among groups, with statistical significance defined as *p* < 0.05 (significant difference) and **p < 0.001 (highly significant difference). The results were shown in Table 2. The coverage of fermented grains samples at different fermentation stages was all 1, indicating that the sequencing depth was sufficient, almost all microbial species in the samples were detected, and the results were reliable. The richness and diversity indices indicated that the Alpha diversity in ZQ was the lowest, suggesting that the microbial community at this stage was dominated by a few dominant species, and the community structure tended to be simplified. The richness and diversity were relatively high in CQ and WQ, but the diversity indices (Shannon and Simpson) were higher in the late stage, indicating that the species distribution was more uniform in the late stage. The results of the Alpha diversity comparative analysis showed that the microbial diversity decreased significantly and the community structure was simplified in ZQ. This was conducive to the efficient metabolism of the dominant microbial community, which was consistent with the previous observation that the metagenomic assembly quality with a high N50 was optimal and the genome assembly of dominant bacteria was more complete in the middle stage. The diversity recovered in WQ and even exceeded that in ZQ, it was hypothesized that the fermentation of WeiJiu was a human-mediated artificial microbial fermentation process, adopting a solid-liquid mixed fermentation mode supplemented by a unique post-fermentation simmering treatment. Its microbial succession was not only affected by interspecific competition but also strictly regulated by the dynamic changes of the brewing microenvironment and the unique fermentation process. The microenvironment of each fermentation stage was directionally constructed around the metabolic needs of brewing sequentially creating specific ecological niches for different functional microorganisms. This directional environmental regulation breaked the “diversity decline” law of natural secondary succession, which was the fundamental reason for the recovery of microbial diversity in the late fermentation stage.

**Table 2:** Results of α-diversity analysis of microbial communities in Congjiang WeiJiu fermented grains at different fermentation stages.

Group	Sample	Observed_species	Chao1	Ace	Shannon	Simpson	Goods_ coverage
CQ	CQ1	58.00 ± 1.53^a^	59.67 ± 2.46^a^	61.45 ± 3.37^a^	1.46 ± 0.00^b^	0.70 ± 0.00^b^	1
	CQ2	55.00 ± 1.53^a^	55.00 ± 2.46^a^	55.30 ± 3.37^a^	1.47 ± 0.00^b^	0.70 ± 0.00^b^	1
	CQ3	56.00 ± 1.53^a^	56.00 ± 2.46^a^	56.00 ± 3.37^a^	1.46 ± 0.00^b^	0.70 ± 0.00^b^	1
ZQ	ZQ1	51.00 ± 2.89^c^	51.25 ± 3.27^c^	52.20 ± 3.56^a^	1.22 ± 0.01^c^	0.60 ± 0.01^c^	1
	ZQ2	51.00 ± 2.89^c^	52.00 ± 3.27^c^	52.14 ± 3.56^a^	1.23 ± 0.01^c^	0.60 ± 0.01^c^	1
	ZQ3	46.00 ± 2.89^c^	46.00 ± 3.27^c^	46.00 ± 3.56^a^	1.20 ± 0.01^c^	0.59 ± 0.01^c^	1
WQ	WQ1	54.00 ± 1.15^a^	57.75 ± 1.88^a^	60.52 ± 2.24^a^	1.53 ± 0.00^a^	0.74 ± 0.00^a^	1
	WQ2	54.00 ± 1.15^a^	56.00 ± 1.88^a^	57.69 ± 2.24^a^	1.53 ± 0.00^a^	0.74 ± 0.00^a^	1
	WQ3	56.00 ± 1.15^a^	59.75 ± 1.88^a^	62.12 ± 2.24^a^	1.52 ± 0.00^a^	0.74 ± 0.00^a^	1
ANOVA	F-value	10.03*	8.68*	8.38*	903.05***	1,513.25***	–
	*p*-value	0.012	0.017	0.018	<0.001	<0.001	–

**Notes:**

CQ, early fermentation stage (7 d); ZQ, middle fermentation stage (11 d); WQ, late fermentation stage (15 d).

The numbers 1–3 indicated three biological replicates. Data were presented as mean ± standard deviation (SD).

Different lowercase letters (a, b, c) in the same column indicated significant differences among groups (Tukey’s HSD *post-hoc* test, *p* < 0.05).

Statistical significance:

* *p* < 0.05,

*** *p* < 0.001;

*** “–” indicated no statistical test performed (Goods_coverage = 1.00 for all samples, indicating sufficient sequencing depth).

### Phylum level microbial structural characteristics and their succession patterns during the fermentation process of WeiJiu

**Figure 1:**
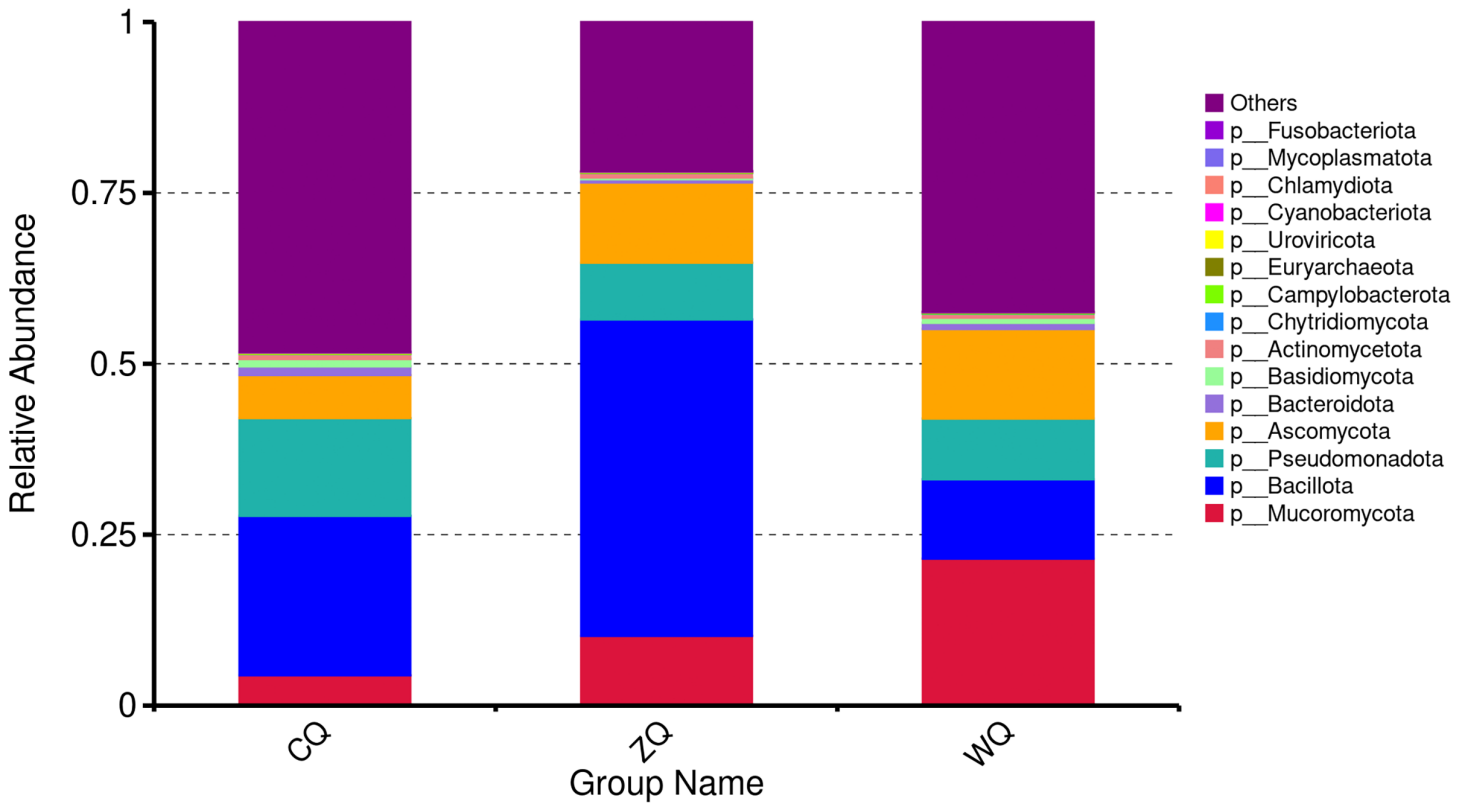
Stacked bar plot of phylum-level relative abundance of microbial communities in Congjiang WeiJiu fermented grains across different fermentation stages. CQ, early fermentation stage (7 d); ZQ, middle fermentation stage (11 d); WQ, late fermentation stage (15 d).

The structural characteristics and succession patterns of the microbial community at the phylum level during the fermentation process of WeiJiu were shown in Fig. 1. At the early fermentation stage, the “Others” group was the absolute dominant community component, with a relative abundance exceeding 50%. This indicated that unclassified microbial taxa accounted for an extremely high proportion at this stage, reflecting the high diversity and complexity of the initial microbial community. This was followed by Bacillota and Pseudomonadota, while the fungal taxa Mucoromycota and Ascomycota each accounted for less than 10% of the total community, indicating an overall bacteria-dominated microbial community at this stage. The relative abundance of the “Others” category decreased significantly, indicating that the microbial community structure became more focused in the middle stage, with distinctly dominant microbial taxa beginning to dominate the fermentation process. In the late fermentation stag, Mucoromycota emerged as the most dominant fungal taxon with a substantial increase in its relative abundance, which suggested its potential involvement in metabolic processes during the maturation phase, such as the biosynthesis of flavor compounds and the degradation of complex substrates. The relative abundance of Bacillota declined, leading to a diminished dominance of bacterial taxa and further expansion of the ecological niches occupied by fungal taxa. Meanwhile, the proportion of the “Others” category increased, and Ascomycota and Pseudomonadota maintained a relatively stable relative abundance, which indicated that the microbial community re-exhibited a trend toward diversification in the late fermentation. In general, the structural characteristics and succession law of microbial community at the phylum level during the fermentative process of WeiJiu were as follows: bacteria predominated in the early stage with an extremely low relative abundance of fungi; the phylum Bacillota was the dominant bacterial group in the middle stage, accompanied by the proliferation of fungi; in the late stage, the phylum Mucoromycota became the dominant fungal group while the relative abundance of bacteria decreased. This fermentation pattern reflected a metabolic division of labor characterized by bacteria-driven initial fermentation and fungus-dominated late maturation.

### Genus-level microbial structural characteristics and their succession patterns during the fermentation process of WeiJiu

**Figure 2:**
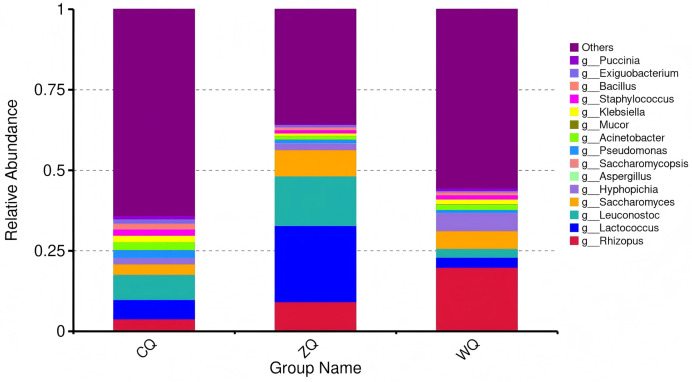
Stacked bar plot of genus-level relative abundance of microbial communities in Congjiang WeiJiu fermented grains across different fermentation stages. CQ, early fermentation stage (7 d); ZQ, middle fermentation stage (11 d); WQ, late fermentation stage (15 d).

An analysis of genus-level microbial community characteristics across different fermentation stages of WeiJiu was presented, with results illustrated in Fig. 2 and the core genera directly involved in WeiJiu brewing and describes their abundance changes across fermentation stages, as well as their specific functional roles in the brewing process were screened in Table 3. In CQ, the microbial community was dominated by the “Others” group with a relative abundance exceeding 75%, which represented a diverse collection of low-abundance bacteria. Only a small proportion of bacterial genera, such as *Lactococcus*, *Acinetobacter*, and *Klebsiella*, were detected. Fungal genera, including *Mucor* and *Saccharomyces*, were present at low levels, confirming that bacteria were the primary drivers of the initial fermentation phase. In ZQ, *Lactococcus* emerged as the most abundant bacterial genus. Fungal genera, particularly *Mucor* and *Saccharomyces*, began to proliferate, indicating a transition to a stage where both bacteria and fungi contributed to metabolic activity. This phase laid the foundation for subsequent flavor compound production. In WQ, fungi became the dominant group, with *Rhizopus* showing the highest relative abundance, followed by *Saccharomyces*. In contrast, the relative abundance of bacterial genera such as *Lactococcus* decreased. This shift to fungal dominance marked the maturation phase, where fungal metabolism played a key role in generating alcohols, esters, and other flavor-active compounds that defined the characteristic taste of WeiJiu.

**Table 3:** Abundance dynamics and functional contributions of brewing-related key functional microbial genera in Congjiang WeiJiu fermented grains at different fermentation stages.

Functional microbial genera	CQ	ZQ	WQ	Functional contribution
*Aspergillus*	High	Moderate	Low	Starch/protein hydrolysis (Pan et al., 2020)
*Saccharomyces*	Low	High	High	Ethanol + CO₂ production (Lodolo et al., 2008).
*Lactococcus*	Moderate	High	Moderate	Maintained an acidic environment (Qiu et al., 2025)
*Hyphopichia*	Moderate	Low	High	Synthesis of ethyl acetate (Cheng et al., 2024)

**Notes:**

CQ, early fermentation stage (7 d); ZQ, middle fermentation stage (11 d); WQ, late fermentation stage (15 d).

The abundance level (high/moderate/low) was determined based on the genus-level relative abundance in Fig. 2.

The functional contributions referred to the core metabolic functions of the genera in the brewing process of fermented wine.

### Dynamic succession trend and functional adaptation mechanism of microbial ommunity during the fermentation process of WeiJiu

**Figure 3:**
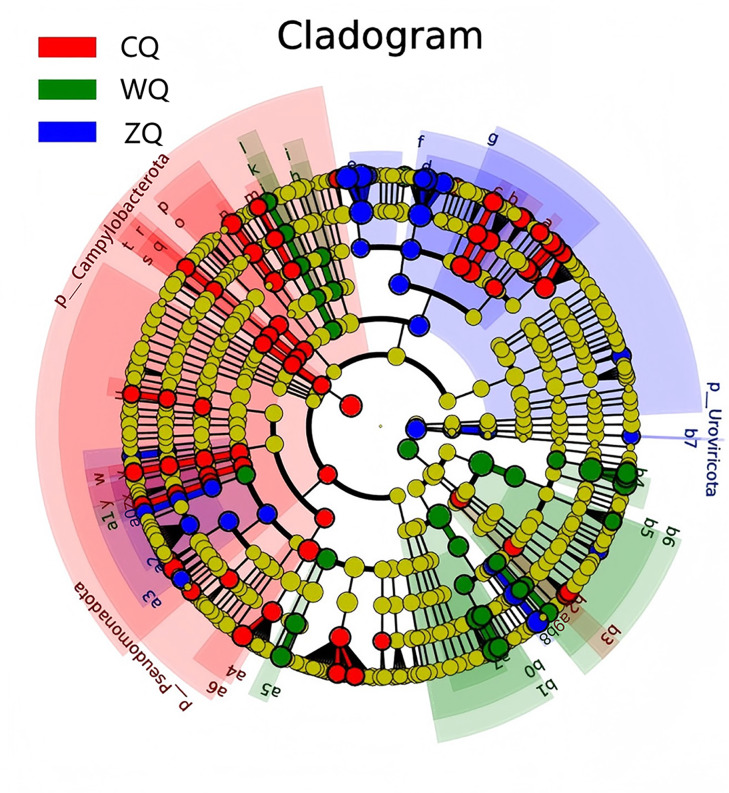
Multi-taxonomic levelphylogenetic cladogram and differential biomarker distribution of microbial communities in Congjiang WeiJiu fermented grains across different fermentation stages. CQ, early fermentation stage (7 d); ZQ, middle fermentation stage (11 d); WQ, late fermentation stage (15 d).

In this study, a microbial phylogenetic tree was constructed to show the dynamic succession of the microbial community during fermentation. The tree was structured radially at the phylum, class, order, family, and genus levels. Phylogenetic relationships were visualized using node diameter (representing relative abundance) and color coding (yellow for non-differential species, red and green for biomarkers in the CQ and WQ groups, respectively) (Fig. 3). Further, through phylogenetic analysis, the differential biomarkers of the microbial communities at each fermentation stage were identified, and their clustering characteristics were clarified. The summary was presented in Tables 4 & 5. *Staphylococcus*, *Escherichia*, and *Acinetobacter*, were identified as the core group in CQ, with long and scattered evolutionary branches. No significant biomarkers were observed in ZQ. In WQ, a dense cluster with short branches was formed, and *Lactobacillus*, *Weissella*, and *Pediococcus*, constituted the core group. It was speculated that in CQ, *Staphylococcus* showed functional advantages in rapid glucose utilization for energy production. In WQ, *Lactobacillus* functioned as the core microorganism, with high efficiency in glucose fermentation and lactic acid production. *Weissella* carried polysaccharide synthesis gene clusters related to dextran production, viscosity increase, phytic acid degradation, and trace element release. These results illustrated the dynamic succession patterns of the microbial community and the functional characteristics of dominant taxa during different stages of WeiJiu fermentation.

**Table 4:** Distribution, taxonomic positions and phylogenetic characteristics of genus-level key differential biomarkers in Congjiang WeiJiu fermented grains at different fermentation stages.

Fermentation stage	Key biomarkers (colored nodes)	Taxonomic position	Phylogenetic characteristics
CQ	*Staphylococcus* *Escherichia* *Acinetobacter*	Bacillota Gammaproteobacteria	Clustered in the gammaproteobacteria glade (dense area of red nodes)
ZQ	Nodes with no significant coloring	No taxonomic status	Transitional Stage, dominant bacteria (*e.g*., *Lactobacilli*) showed dispersed distribution on the phylogenetic tree
WQ	*Lactobacillus* *Weissella* *Pediococcus*	Bacillota Bacilli	Concentrated in the Lactobacillales order clusters (dense area of green nodes)

**Notes:**

CQ, early fermentation stage (7 d); ZQ, middle fermentation stage (11 d); WQ, late fermentation stage (15 d).

Key biomarkers were identified from the differential taxa in Fig. 3. Phylogenetic characteristics were summarized based on the clustering pattern of biomarker taxa in the phylogenetic tree of Fig. 3.

**Table 5:** Analysis of functional advantages and evolutionary adaptation mechanisms of key biomarkers in Congjiang WeiJiu fermented grains at different fermentation stages.

Biomarker	Corresponding fermentation stage	Functional advantage	Evolutionary adaptation mechanism
*Staphylococcus*	CQ	Rapid utilization of glucose for energy production	Carrying multiple antibiotic-resistance genes (acquired through horizontal gene transfer (Vitko et al., 2016; Zeng et al., 2024; Savage, Chopra & O’Neill, 2013)
*Lactobacillales*	WQ	Efficient glycolysis for lactic acid production; Synthesizing bacteriocins to inhibit miscellaneous bacteria	Genome reduction (loss of non-essential genes) and enhancement of core metabolism (Xue, 2023; Feyereisen et al., 2020)
*Weissella*	WQ	Synthesizing dextran to enhance wine viscosity; Degrading phytate to release trace elements	Obtaining exogenous polysaccharide synthesis gene cluster (Koirala et al., 2021; Xiang et al., 2023)

**Note:**

CQ, early fermentation stage (7 d); ZQ, middle fermentation stage (11 d); WQ, late fermentation stage (15 d).

### KEGG functional analysis at different fermentation stages

**Figure 4:**
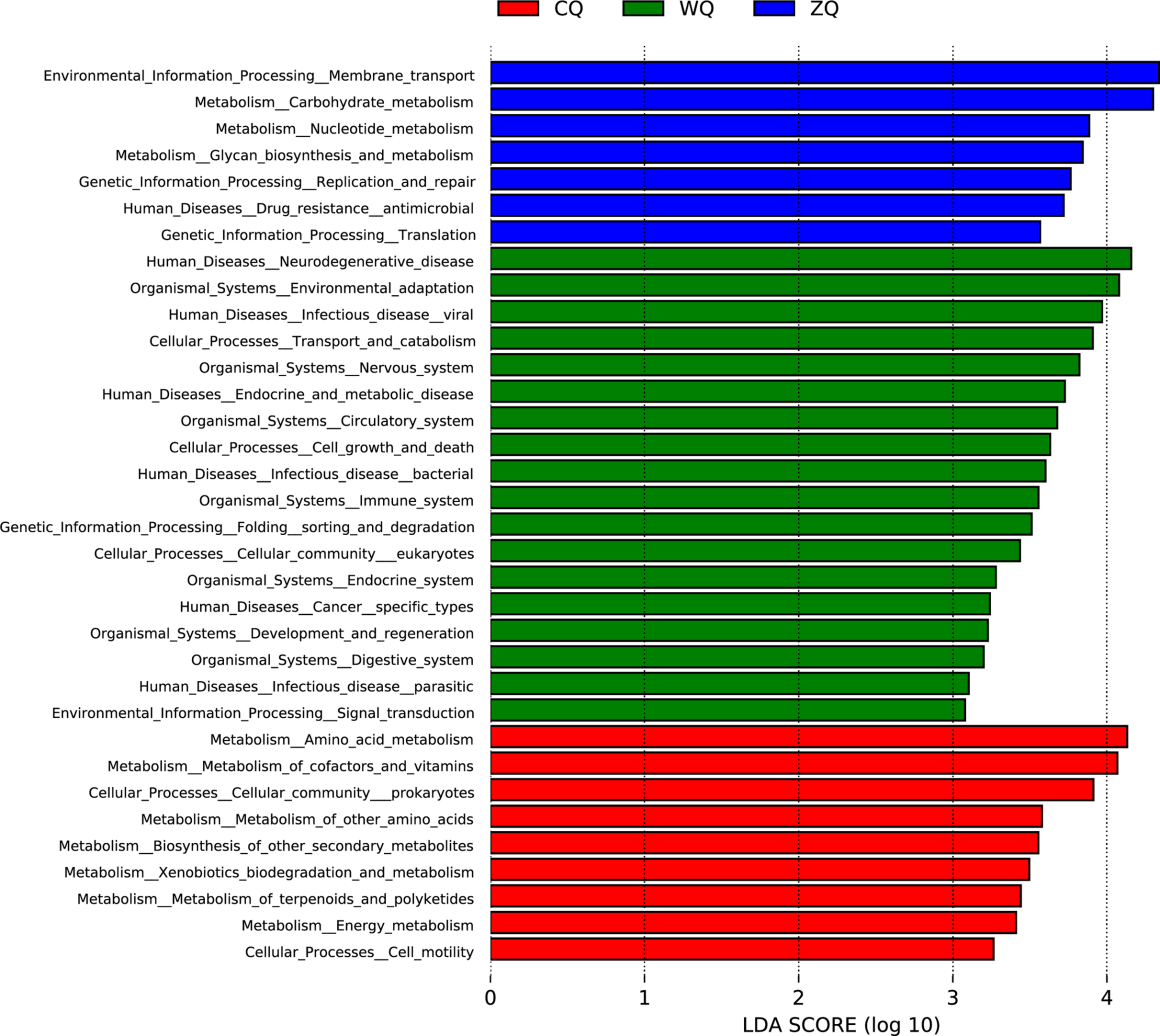
LDA score distribution plot of KEGG functional pathways in microbial communities of Congjiang WeiJiu fermented grains across different fermentation stages. CQ, early fermentation stage (7 d); ZQ, middle fermentation stage (11 d); WQ, late fermentation stage (15 d), sorted by enrichment level.

KEGG functional analysis was performed using LEfSe (LDA Effect Size). Genes were annotated to determine functional abundance, and statistical tests were conducted to characterize metabolic pathways during fermentation (Fig. 4). The results showed that KEGG functions with high LDA scores in CQ were mainly associated with metabolic pathways, including amino acid metabolism, metabolism of cofactors and vitamins, and energy metabolism. In ZQ, the diversity of KEGG functions was notably increased, covering carbohydrate metabolism, nucleotide metabolism, genetic information processing, and environmental information processing. In WQ, the profile of KEGG functions shifted markedly, with predominant enrichment in pathways related to human diseases, organismal systems and cellular processes.

### CAZys functional analysis at different fermentation stages

**Figure 5:**
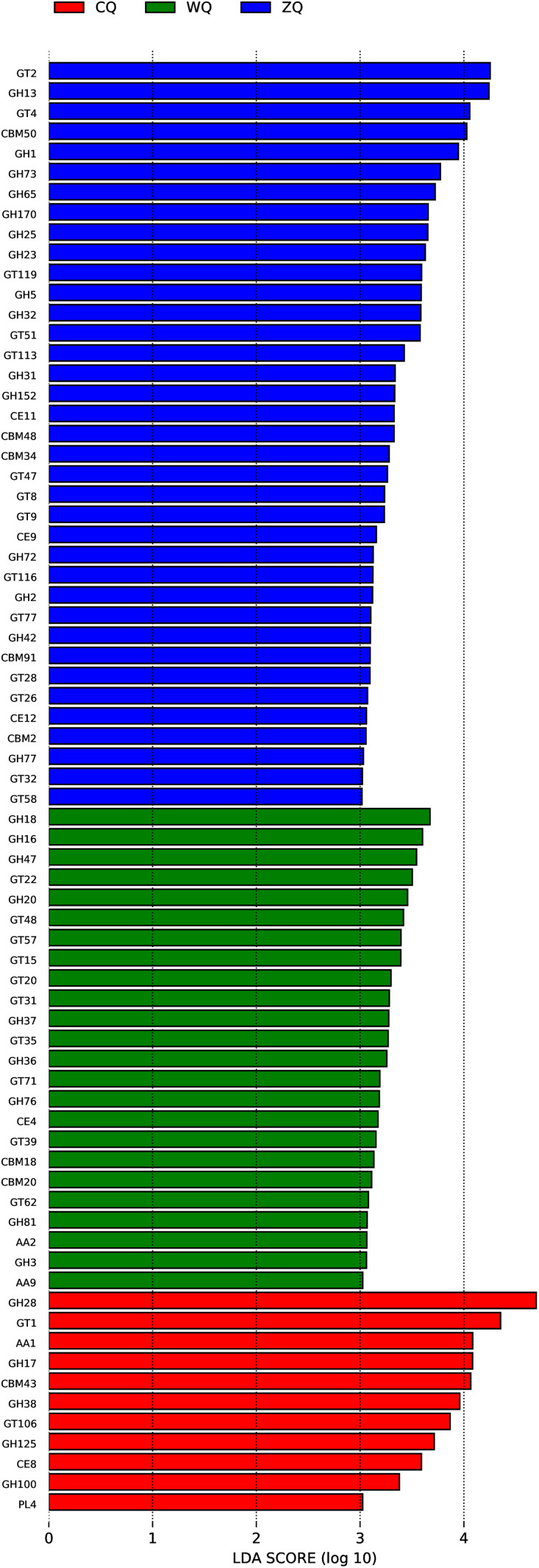
LDA score distribution plot of CAZys functional families in microbial communities of Congjiang WeiJiu fermented grains across different fermentation stages. CQ, early fermentation stage (7 d); ZQ, middle fermentation stage (11 d); WQ, late fermentation stage (15 d), sorted by enrichment level. In the CAZys database, the prefixes before the classification names of various families represent the meanings as follows: GH, Glycoside Hydrolase; GT, Glycosyltransferase; CBM, Carbohydrate-Binding Module; CE, Carbohydrate Esterase; AA, Auxiliary Activity; PL, Polysaccharide Lyase. The numbers following the acronyms (*e.g.*, “13” in “GH13”) represented the specific family number of each CAZys category in the CAZys Database.

Studies have demonstrated that carbohydrate-active enzymes (CAZy enzymes) played a crucial role in the fermentation process of traditional alcoholic beverages, with their functions closely linked to flavor formation and product quality (Ma et al., 2024). Variations in CAZy enzyme profiles across fermentation stages reflect the complexity of the process. To investigate these differences during WeiJiu fermentation, LDA Effect Size (LEfSe) analysis was employed to identify microbial CAZy functional enzymes that were significantly enriched at each stage (Fig. 5). In CQ, CAZy functions with high LDA values were concentrated in specific gene families, including GH28, GT1, and AA1. In ZQ, CAZy functional diversity increased significantly, and high LDA values were observed for multiple gene families such as GT2, GH13, GT4, and CBM50. In WQ, the distribution of CAZy functions altered notably, with the main enrichment in GH18, GH16, and GH47 gene families.

## Discussion

### Synergistic adaptation between microbial community succession and fermentation stages

The microbial community of WeiJiu fermented grains showed obvious stage-specific succession characteristics during the fermentation process, and this succession was highly synergistic with the environmental changes and metabolic requirements of each fermentation stage, which was a typical manifestation of the co-evolution of microbial community and fermentation microenvironment. This succession pattern shared the common characteristics of microbial adaptation in traditional Chinese Baijiu fermentation, and also formed its own uniqueness due to the particularity of WeiJiu’s brewing process.

At the phylum level, the fermentation early stage was dominated by Pseudomonadota with the high relative abundance. These aerobic/facultative anaerobic microorganisms could rapidly consume the oxygen and simple carbohydrates in the fermented grains microenvironment, which was the key to the rapid establishment of an anaerobic environment required for subsequent fermentation and was consistent with the early aerobe-driven environmental preparation pattern found in the fermentation of traditional fermented alcoholic beverages (Bokulich et al., 2014). Entering the middle fermentation stage, Bacillota replaced Pseudomonadota as the core microbial phylum. The relative abundance of Bacillota in ZQ of WeiJiu was about 60%, which was significantly higher than that of traditional Fen-flavor Baijiu. This unique characteristic was speculated to be closely related to the specific microbial strains introduced by the traditional homemade *koji* in the spreading, cooling and *koji* mixing stage, which was one of the important reasons for the difference between WeiJiu and other Baijiu in microbial community structure. In the late fermentation stage, the relative abundance of Actinomycetota increased. This phylum of microorganisms was good at synthesizing complex secondary metabolites such as esters and terpenes, and could synergize with the ester-producing capacity of Hyphopichia to jointly promote the finalization of WeiJiu’s characteristic flavor. This co-metabolism mode for flavor enhancement was similar to the Actinobacteria-yeast synergistic mechanism in the late fermentation stage of Huangjiu (Mao, 2020). In addition, Euryarchaeota and Campylobacterota were detected in the fermented grains of WeiJiu, which were rarely found in other traditional fermented alcoholic beverages. It was hypothesized that these rare microbial phyla might be involved in the degradation of residual organic impurities in the late fermentation stage and the production of special flavor compounds, and their specific functional mechanisms need to be further verified by combining metabolomics data in subsequent studies. Notably, Cyanobacteriota was detected as a low-abundance phylum in the fermented grains across all fermentation stages, which was consistent with the occasional occurrence of Cyanobacteria in starch-based fermented systems (Wan et al., 2023; Yang et al., 2024). We infered that the core raw material of WeiJiu was local fragrant glutinous rice and mountain spring water. Cyanobacteria were widely distributed in aquatic environments and soil, and could adhere to the surface of rice grains during cultivation or survive in unpasteurized mountain spring water (Ji et al., 2018). During raw material preparation, these epiphytic or aquatic Cyanobacteria might be introduced into the fermentation system, forming the low-abundance colonization.

At the genus level, the dynamic changes of core functional microorganisms directly reflected the stage-specific functional requirements of WeiJiu fermentation. In CQ, *Aspergillus* played a core role in the initial saccharification and proteolysis process, and the amylolytic enzyme and proteolytic enzyme systems it secreted efficiently hydrolyzed the starch and protein in fermentation substrates into small molecular nutrients such as monosaccharides and amino acids, ensuring the continuous supply of carbon and nitrogen sources for the subsequent microbial metabolism, which was consistent with the conclusion that *Aspergillus* dominated the saccharification initiation stage in the fermentation of Special-flavored Baijiu (Pan et al., 2020). In ZQ, *Saccharomyces and Lactococcus* underwent explosive growth and became the core microorganism. *Saccharomyces* leaded to a significant increase in ethanol content in the fermented grains, and the large amount of CO₂ released during its metabolism also further optimized the anaerobic fermentation environment, which was in line with the core alcohol-producing function of *Saccharomyces* in the alcohol-acid conversion stage of traditional fermentation (Lodolo et al., 2008; Albergaria & Arneborg, 2016; Krogerus & Gibson, 2020). *Lactococcus* produced lactic acid through homolactic fermentation, which not only lowers the fermentation pH to protect *Saccharomyces* from contamination but also provided a mild acidic condition that promoted the activity of alcohol dehydrogenase in *Saccharomyces*, enhancing ethanol synthesis efficiency. This synergistic metabolic mechanism was consistent with the “*Lactobacillus*-Yeast” collaboration reported in Luzhou-flavor liquor fermentation (Hu et al., 2020; Qiu et al., 2025), In WQ, *Hyphopichia* became the dominant ester-producing microorganism, and its secreted esterase catalyzed the key synthesis reaction of ethyl acetate, which was the core substance forming the mellow, soft, sweet and fragrant characteristic flavor of WeiJiu. This made *Hyphopichia* a key microbial marker distinguishing WeiJiu from other traditional fermented alcoholic beverages. Previously, the ester-producing potential of *Hyphopichia* was only found in Awati red grape wine (Cheng et al., 2024), and this study confirmed for the first time its core functional role in the fermentation of traditional rice wine, enriching the research on the ester-producing microbial resources of traditional fermented alcoholic beverages.

The α-diversity analysis results further revealed the ecological adaptation strategy of the microbial community during WeiJiu fermentation: the microbial diversity was the lowest in ZQ, and no significant biomarker microorganisms were detected, forming a typical “diversity valley” phenomenon. This was a key ecological node for the optimization of the microbial community structure of WeiJiu fermented grains-dominant microorganisms such as *Lactococcus* and *Saccharomyces* occupied the main ecological niches through functional redundancy, which was conducive to the concentration of microbial metabolic activities and the maximization of fermentation efficiency such as lactic acid and ethanol production. In WQ, the microbial diversity recovered and even exceeded that in CQ, which was closely related to the accumulation of secondary metabolites in the late fermentation stage and the microbial demand for the utilization of residual nutrients. The enrichment of functional microorganisms such as *Weissella* and *Lactobacillus* further enriched the functional diversity of the community, and the improvement of microbial diversity provided a rich metabolic basis for the formation of complex flavor substances of WeiJiu, which was consistent with the oenological consensus that late-stage microbial diversity supported the flavor complexity of fermented alcoholic beverages (Jia et al., 2024; Xiang et al., 2023).

### Stage-specific expression of microbial functional characteristics and metabolic pathways

In terms of KEGG functional characteristics, the microbial metabolism in CQ was mainly concentrated in amino acid metabolism, cofactor and vitamin metabolism, and energy metabolism pathways. These core metabolic pathways provided sufficient material and energy basis for microorganisms to adapt to the new fermentation environment with continuous changes in temperature, oxygen and nutrient conditions. Among them, amino acid metabolism could generate branched-chain alcohol precursors through the Ehrlich pathway, laying a foundation for the subsequent formation of flavor alcohols, and energy metabolism could rapidly decompose glucose to provide energy for microbial colonization and proliferation, which was consistent with the research conclusion that early-stage microbial metabolism was mainly oriented to self-growth and environmental adaptation (Clement et al., 2013). In ZQ, the diversity of microbial metabolic pathways increased significantly, and carbohydrate metabolism, nucleotide metabolism, membrane transport and genetic information processing pathways were significantly enriched. The enhanced membrane transport function could effectively promote the microbial absorption of carbohydrates, amino acids and other nutrients, and accelerate the excretion of metabolic products such as ethanol, avoiding the toxic effect of high concentration of ethanol on microorganisms. Carbohydrate metabolism and nucleotide metabolism were the core pathways supporting ethanol synthesis, and the increased flux of glycolysis pathway was the key reason for the peak of ethanol production in ZQ, which was consistent with the conclusion of metabolic flux analysis that middle-stage core carbon metabolism dominated alcohol production (Quirós et al., 2013). In WQ, the microbial metabolic profile showed a significant shift, and the functions related to human diseases and cellular processes became the dominant pathways. Although these functional categories seemed to have no direct correlation with the core fermentation process such as ethanol and lactic acid synthesis, they essentially reflected the adaptive metabolic strategies of microorganisms to the stressful fermentation environment in the late stage (nutrient depletion, high ethanol concentration, low pH). Specifically, the functions related to neurodegenerative diseases were corresponding to the protein degradation and recycling process of microbial cells under environmental stress, and the functions related to infectious diseases might be associated with the microbial competition and inhibition of miscellaneous bacteria through metabolic regulation. This stress response mechanism was similar to the late-stage cellular stress promoting microbial autolysis and flavor product release found in sparkling wine yeast fermentation (Juan Antonio et al., 2019). In addition, the enhancement of environmental adaptation and immune system-related functions in WQ further ensured the stability of the microbial community structure under adverse conditions, providing a reliable guarantee for the continuous accumulation and modification of flavor substances.

At the CAZys functional level, the stage-specific expression of carbohydrate-active enzyme gene families directly reflected the dynamic process of microbial degradation and transformation of carbohydrates in fermentation substrates during WeiJiu fermentation. In CQ, GH28, GT1 and AA1 gene families were significantly enriched: GH28 could efficiently decompose pectin in fermentation substrates, providing initial carbon sources for microbial growth; AA1 could assist microorganisms to release the internal nutrients of fermentation substrates, which was consistent with the research result that GH28 played a key role in pectin degradation in the early stage of fermentation (Li et al., 2017). The synergistic effect of these enzyme families laid a foundation for the subsequent in-depth utilization of fermentation substrates by microorganisms. In ZQ, GH13, GT2/GT4 and CBM50 gene families became the dominant CAZys functions: GH13 as the core enzyme system for starch degradation, its high activity indicated that ZQ was the core stage for the degradation of macromolecular carbohydrates such as starch in WeiJiu fermentation, which was consistent with the conclusion that GH13 supported the efficient conversion of starch in traditional fermented alcoholic beverages (Duan & Wu, 2013); CBM50 could improve the binding efficiency of enzymes and carbohydrate substrates, further enhancing the degradation efficiency of macromolecular carbohydrates (Wei et al., 2022). The synergistic action of these enzyme families promoted the rapid conversion of fermentation substrates carbohydrates into fermentable monosaccharides, providing sufficient carbon sources for ethanol synthesis. In WQ, GH18, GH16 and GH47 gene families were the main enriched CAZys functions: GH18 could degrade nitrogen-containing carbohydrates such as chitin, effectively supplementing the nitrogen source deficiency in the late fermentation stage and ensuring the normal metabolic activity of microorganisms; GH16 and GH47 could modify the structure of metabolic products such as glycosides, directly participating in the modification and formation of flavor substances (Mouyna et al., 2016; Liberato et al., 2021; Quirke & Crich, 2021). The stage-specific division of CAZys functions in WeiJiu fermentation was highly consistent with the universal metabolic logic of traditional brewing-early raw material degradation, middle product synthesis, late flavor modification (Geng et al., 2021), which was the result of the long-term adaptation of microbial community to the brewing process of WeiJiu.

### Supporting role of microbial evolutionary adaptation mechanisms in fermentation functions

In CQ, *Staphylococcus*, as the core functional bacterium, had formed two key evolutionary adaptation mechanisms: on the one hand, it acquired multiple antibiotic resistance genes through horizontal gene transfer, which significantly enhanced its survival competitiveness in the complex microbial community environment of the early fermentation stage with diverse microorganisms; on the other hand, it had the functional advantage of rapid utilization of glucose for energy production, which could quickly provide energy for the early preparation of the fermentation environment. This evolutionary adaptation mechanism of *Staphylococcus* was consistent with the research conclusion that horizontal gene transfer could improve the carbon source utilization efficiency of *Staphylococcus* aureus (Vitko et al., 2016; Savage, Chopra & O’Neill, 2013). In addition, *Staphylococcus* still maintained a certain relative abundance in ZQ, which indicated that it was not only the core functional bacterium for the initial environmental preparation, but also participated in the middle fermentation metabolism, and was a key functional microorganism throughout the early and middle fermentation stages of WeiJiu. In WQ, It was infered *Lactobacillus* became the dominant core functional bacterium by virtue of the genome streamlining evolutionary mechanism: it deleted non-essential genes in the genome evolution process, and further strengthened the key metabolic pathways closely related to fermentation functions such as glycolysis and bacteriocin synthesis. This mechanism made *Lactobacillus* adapt to the low pH environment in the late fermentation stage with a relative abundance of more than 70%, and its efficient lactic acid production capacity could maintain the acidic microenvironment of fermented grains, and the bacteriocin it synthesizes could further inhibit the growth of miscellaneous bacteria, thus maintaining the stability of the microbial community structure in WQ. This evolutionary adaptation characteristic was consistent with the research result that genome streamlining can significantly enhance the environmental stress resistance of *Lactobacillus* (Feyereisen et al., 2020), and the dominant position of *Lactobacillus* in WQ was the core guarantee for the stability of the fermentation process and the formation of flavor substances in the late stage of WeiJiu. *Weissella*, as an important functional bacterium in WQ, had acquired exogenous polysaccharide synthesis gene clusters through plasmid-mediated horizontal gene transfer, which endowed it with unique dual metabolic functions: on the one hand, it synthesized dextran to increase the viscosity of the wine body, forming the mellow and soft taste characteristic of WeiJiu; on the other hand, it degraded phytic acid in the fermentation substrates to release trace elements, enriching the nutritional characteristics of WeiJiu. Previously, the dextran synthesis potential of *Weissella* was only found in the fermentation of beer by-products (Koirala et al., 2021), and this study was the first to discover its multiple metabolic functions in traditional rice wine fermentation. This unique functional characteristic of *Weissella* was speculated to be the result of the long-term domestication of the microbial community by the traditional homemade *koji* and unique simmering process of WeiJiu, which enriched the research on the evolutionary adaptation and functional characteristics of *Weissella* in traditional fermented alcoholic beverages.

## Conclusions

In conclusion, this study took the fermented grains at different fermentation stages of Congjiang WeiJiu as the research object, used high-throughput sequencing technology to systematically analyze the microbial community structure and functional characteristics, clarified the synergistic adaptation relationship between microbial community succession and fermentation process, and revealed the stage-specific expression characteristics of microbial core functions and metabolic pathways, as well as the evolutionary adaptation mechanisms of core functional microorganisms. The research results filled the research gap of microbial mechanism in the brewing process of WeiJiu, and provided an important theoretical basis for the scientific interpretation and upgrading of traditional brewing technology, the improvement of product quality and the realization of industrialized production. At the same time, the research also enriched the microbial resource database and functional mechanism research of traditional ethnic fermented alcoholic beverages in China, and provided a reference for the protection and development of other intangible cultural heritage brewing techniques.

## Additional Information and Declarations

### Data Availability

The following information was supplied regarding data availability:

The data is available at NCBI SRA: PRJNA1372463, PRJNA1372792, PRJNA1372643.
